# Health-related quality of life in different trimesters during pregnancy

**DOI:** 10.1186/s12955-021-01811-y

**Published:** 2021-07-21

**Authors:** Huailiang Wu, Weiwei Sun, Hanqing Chen, Yanxin Wu, Wenjing Ding, Shangqiang Liang, Xinyu Huang, Haitian Chen, Qing Zeng, Zhuyu Li, Peng Xiong, Jian Huang, Babatunde Akinwunmi, Casper J. P. Zhang, Wai-Kit Ming

**Affiliations:** 1 Department of Public Health and Preventive Medicine, School of Medicine, Jinan University, Guangzhou, China; 2 Faculty of Medicine, International School, Jinan University, Guangzhou, China; 3 Department of Obstetrics and Gynaecology, The First Affiliated Hospital of Sun Yat-Sen University, Guangzhou, Guangdong China; 4 Department of Epidemiology and Biostatistics, School of Public Health, Imperial College London, London, UK; 5 Maternal-Fetal Medicine Unit, Department of Obstetrics and Gynecology, Brigham and Women’s Hospital, Boston, USA; 6 Center for Genomic Medicine (CGM), Massachusetts General Hospital, Harvard Medical School, Harvard University, Boston, MA USA; 7 School of Public Health, The University of Hong Kong, Hong Kong, China; 8 Singapore Institute for Clinical Sciences (SICS), Agency for Science, Technology and Research (A*STAR), Singapore, Singapore

**Keywords:** Health-related quality of life, HRQoL, Pregnancy, EQ-5D-5L, Pregnant women, Trimesters

## Abstract

**Background:**

Pregnant women experience physical, physiological, and mental changes. Health-related quality of life (HRQoL) is a relevant indicator of psychological and physical behaviours, changing over the course of pregnancy. This study aims to assess HRQoL of pregnant women during different stages of pregnancy.

**Methods:**

This cross-sectional study was performed using the The EuroQoL Group’s five-dimension five-level questionnaire (EQ-5D-5L) to assess the HRQoL of pregnant women, and demographic data were collected. This study was conducted in a regional university hospital in Guangzhou, China.

**Results:**

A total of 908 pregnant women were included in this study. Pregnant women in the early 2^nd^ trimester had the highest HRQoL. The HRQoL of pregnant women rose from the 1st trimester to the early 2nd trimester, and dropped to the bottom at the late 3rd trimester due to some physical and mental changes. Reports of pain/discomfort problem were the most common (46.0%) while self-care were the least concern. More than 10% of pregnant women in the 1st trimester had health-related problems in at least one dimension of whole five dimensions. In the whole sample, the EuroQoL Group’s visual analog scale (EQ-VAS) was 87.86 ± 9.16. Across the gestational stages, the HRQoL remained stable during the pregnancy but the highest value was observed in the 1st trimester (89.65 ± 10.13) while the lowest was in the late 3rd trimester (87.28 ± 9.13).

**Conclusions:**

During pregnancy, HRQoL were associated with gestational trimesters in a certain degree. HRQoL was the highest in the early 2nd trimester and then decreased to the lowest in the late 3rd trimester due to a series of physical and psychological changes. Therefore, obstetric doctors and medical institutions should give more attention and care to pregnant women in the late 3rd trimester.

## Background

Pregnant women experience physical, physiological, and mental changes. In general, pregnancy is an exciting and desired event, but it also involves considerable inconvenience, discomfort, and sometimes mood changes or depression due to various physical and physiological changes [1]. These changes tend to increase with time and can significantly affect psychological and physical conditions of pregnancy women in different ways. For example, a larger uterus can cause difficulty with movement or an increased cardiac load which can lead to chest tightness [2]. Therefore, physical and mental health states of pregnant women change over the course of pregnancy. With the increasing focus on mental health, obstetricians have increasingly assessed the psychological status of pregnant women.

The quality of life (QoL) of an individual as defined by the World Health Organization (WHO) is said to be the person’s general well-being including mental status, stress level, sexual function and self-perceived health status. The Health-related Quality of life (HRQoL) of an individual encompasses mental health status, physical well-being, psychological well-being and is also a relevant indicator of psychological and physical behaviors [3]. The EuroQoL Group’s five-dimension five-level questionnaire (EQ-5D-5L), a reliable instrument developed by the Euro-QoL group, includes a descriptive and evaluative portion that measures health-related quality of life (HRQoL) [4, 5]. Subjects assess the state of their overall health using the EuroQoL Group’s visual analog scale questionnaire (EQ-VAS) in the evaluative section and using five dimensions (mobility, self-care, usual activities, pain/discomfort, anxiety/depression) in the descriptive section [6]. The severity of these five dimensions is quantified using a five-level rating scale [6]. The EQ-5D-5L questionnaire has been frequently used to assess the HRQoL of various populations, ranging from general population to patients with mental disorders, cancers, etc. [7–11].

The World Health Organization reported that 10% of pregnant women who have recently given birth experienced psychological problems, the most common being depression [12]. To better understand the health of pregnant women, HRQoL is increasingly considered as an important indicator that assesses these women’s physical and psychological health. Studies have suggested that prenatal anxiety/depression and/or fear of childbirth could affect the HRQoL of pregnant women [13, 14]. Issues with body image, excessive weight gain, and loss of sexual function during pregnancy all contribute to antepartum depression [15, 16]. Furthermore, complications such as gestational diabetes mellitus and preeclampsia can negatively influence HRQoL, despite most of these complications being short-term and reversible [17–19]. Sut et al. found that pregnancy was significantly related to a more negative HRQoL [20]. Campolong et al. [21] reported that women who received sufficient exercises during pregnancy had a better HRQoL than women who did not meet the recommended guidelines for physical activities.

Further formulation of health policies and clinical guidelines associated with pregnancy requires the analysis of health economics and normative values of HRQoL during pregnancy [22]. However, to the best of our knowledge, the relationship between HRQoL and gestational age changes has not been investigated. The aim of this study was to assess the HRQoL of pregnant women and how HRQoL changes during pregnancy. This investigation can provide insight into improving quality of life of pregnant women and supporting health policies in the future.

## Objectives

We aimed to evaluate several parameters: (1) determine the HRQoL in pregnant women with different gestational ages; (2) provide a utility-based case value in different gestational ages.

## Methods

### Study population

We performed a cross-sectional study of pregnant women who received antenatal care at the First Affiliated Hospital of Sun Yat-sen University, a regional teaching hospital in South China. Ethnically Chinese women with live pregnancy were recruited from June 2016 to October 2018 in this study. One of the research assistants invited each potentially eligible participant and explained the objectives, procedure, risks and benefits of our study. Upon verification of eligibility and provision of written informed consent, participants were asked to complete the questionnaires during checking in at the antenatal clinics during June 2016 to October 2018. We have collected 1571 questionnaires totally. Inclusion criteria: (1) ethnically Chinese women with live pregnancy; (2) attended antenatal care sessions in the First Affiliated Hospital of Sun Yat-sen University between June 2016 to October 2018. Exclusion criteria: (1) participants had missing demographic information and/or clinical data; (2) participants did not complete the questionnaires for the first time. Data of this study was based on questionnaire survey conducted by sequential sampling of patients in antenatal clinics and obstetrics inpatient department. Pregnant women met the inclusion criteria and agreed to participate were included in this study. There were 11 questionnaires to be excluded because of missing data and 652 questionnaires to be excluded because they were not filled out for the first time by participants. Finally, 908 pregnant women were included in the analysis and there were 49, 289 and 570 participants in the first, second and third trimesters respectively.

### Variables

Demographic data and health status (pregnant status, cardiovascular diseases, hepatitis B, gestational diabetes mellitus, scarred uterus and complications) of the pregnant women and their partners were collected in the questionnaires. Pre-pregnancy body mass index (BMI) of the participants was calculated using body weight (kilograms) before pregnancy and height (meters) obtained from electronic medical records system. No study subjects were pregnant for more than 42 gestational weeks.

Participants were requested to fill in the EQ-5D-5L questionnaire when they attended antenatal care sessions in the First Affiliated Hospital of Sun Yat-sen University. Self-reported indications measure the severity experienced by the participants in the five dimensions, i.e., mobility, self-care, usual activities, pain/discomfort, anxiety/depression. Each dimension was evaluated using a 5-level scale: extreme/unable (level 5), severe (level 4), moderate (level 3), slight (level 2), and none (level 1). For example, a response of ‘1,1,2,2,1’ indicates the participant has no problem walking (level 1 of mobility) or washing or dressing (level 1 of self-care), slight problems in work or study (level 2 of usual activities), slight pain or discomfort (level 2 of pain/discomfort), and no anxiety or depression (level 1 of anxiety/depression). A dichotomous variable can be defined for each dimension based on the EQ-5D-5L questionnaire, i.e., ‘have problem’ (levels 2–5) and ‘no problem’ (level 1).

To estimate the HROoL score, we aggregated the five dimensions used the EQ-5D-5L Crosswalk Index Value Calculator [6]. Specifically, a weight was assigned in the EQ-5D-5L questionnaire. A weight equals to 1 indicates “full health”, a weight equals to 0 indicates “dead”, and a weight of -0.224 indicates the participants consider the condition is worse than death. The index value of a certain health state can be obtained by subtracting the corresponding weight of the health state in each dimension from 1 (that is, the utility index value of the complete health state 11111).

We also estimated the EQ-VAS scores, which records the respondent’s self-rated health on a vertical, visual analogue scale with endpoints labelled “the best health you can imagine” and “the worst health you can imagine”, and it can be used as a quantitative measure of health as judged by the individual respondents [6]. Pregnant women self-evaluated their general health status, with 0 being the lowest (the worst potential health status) and 100 being the highest (the best potential health status) [23]. The EQ index value is calculated from the EQ-5D-5L descriptive system via the “EQ-5D-5L Crosswalk Index Value Calculator”.

EQ-5D-5L was proved to show good validity and reliability in previous studies [24, 25]. The Chinese version of the EQ-5D-5L has been proved to be valid and effective that is commonly used to measure HRQoL [26–28]. And this dimension-based value can also facilitate the calculation of quality-adjusted life years, which are used to inform economic evaluations of health care intervention [6].

### Statistical analysis

The EQ-5D-5L index value was calculated using the EQ-5D-5L Crosswalk Index Value Calculator [6]. The algorithm was developed from a general Japanese sample using time trade-off valuation techniques. All statistical analysis was performed using SPSS version 20.0 (SPSS Inc., Chicago, IL, USA). Normally distributed continuous variables were expressed as means ± standard deviation (SD); non-normal variables were presented as median (interquartile range, IQR), and categorical variables were presented as a number and percentage. The One-Way Analysis of Variance (ANOVA) test was used to calculate the demographic data of pregnant women in five gestational stages in Table 1 and the EQ-VAS and EQ index value of pregnant women in different conditions cross five gestational stages in Table 2. A Chi-square test was used to analyze the reporting levels from 1 to 5 in EQ-5D dimensions of pregnant women in different conditions across five gestational stages in Table 3.

**Table 1:** Baseline characteristics of the study sample (n = 908)

	1st	Early 2nd	Late 2nd	Early 3rd	Late 3rd	Overall	*P* value
	Mean (SD)	Mean (SD)	Mean (SD)	Mean (SD)	Mean (SD)	Mean (SD)	
Age	34.41 (4.76)	35.9 (4.95)	35.59 (4.67)	35.49 (5.05)	36.43 (4.75)	35.84 (4.86)	0.029*
BMI	22.76 (4.01)	22.15 (3.45)	23.52 (4.01)	24.36 (4.07)	26.8 (26.31)	24.70 (16.08)	0.039*
Gravidity	1.04 (0.93)	1.16 (0.98)	1.18 (1.11)	1.23 (1.14)	1.2 (1.16)	1.19 (1.11)	0.855
Parity	0.61 (0.57)	0.56 (0.55)	0.52 (0.58)	0.54 (0.52)	0.5 (0.51)	0.53 (0.53)	0.583
EQ-VAS^a^	89.65 (10.13)	87.38 (9.38)	88.32 (8.95)	88.17 (9.03)	87.28 (9.13)	87.86 (9.16)	0.377
EQ index value^b^	0.79 (0.31)	0.89 (0.12)	0.86 (0.13)	0.83 (0.19)	0.82 (0.15)	0.84 (0.17)	< 0.001*

One-Way Analysis of Variance (ANOVA) test was used to calculated the data

SD = standard deviation

*P value < 0.05 indicates the statistical difference

^a^EuroQol-visual analogue scale

^b^EuroQol index value

**Table 2:** EQ-VAS, EQ index value and reported problems (percentage) in EQ-5D dimensions of pregnant women in different conditions cross five gestational stages

Condition	Trimester-specific					Overall	*P* value
	1st	Early 2nd	Late 2nd	Early 3rd	Late 3rd		
*Singleton*	n = 48	n = 107	n = 171	n = 234	n = 313	n = 873	
EQ-VAS^a^	89.44 (10.12)	87.33 (9.35)	88.31 (8.99)	88.4 (8.85)	87.25 (9.05)	87.9 (9.09)	0.347
EQ index value^b^	0.79 (0.31)	0.89 (0.12)	0.87 (0.13)	0.84 (0.19)	0.82 (0.15)	0.84 (0.17)	< 0.001*
Mobility	12 (25.0)	8 (7.5)	16 (9.4)	49 (20.9)	76 (24.3)	161 (18.4)	< 0.001*
Self-care	7 (14.6)	1 (0.9)	9 (5.3)	26 (11.1)	40 (12.8)	83 (9.5)	< 0.001*
Usual activity	9 (18.8)	5 (4.7)	23 (13.5)	43 (18.4)	65 (20.8)	145 (16.6)	0.002*
Pain/discomfort	41 (85.4)	41 (38.3)	70 (40.9)	96 (41.0)	167 (53.3)	415 (47.5)	< 0.001*
Anxiety/depression	28 (58.3)	28 (26.2)	50 (29.2)	60 (25.6)	103 (33.0)	269 (30.8)	< 0.001*
*Multiple pregancy*	n = 0	n = 7	n = 2	n = 10	n = 8	n = 27	
EQ-VAS^a^	–	88.14 (10.61)	92.5 (10.61)	81.5 (11.07)	86.88 (13.61)	85.63 (11.62)	–
EQ index value^b^	–	0.87 (0.13)	0.63 (0.06)	0.72 (0.15)	0.69 (0.09)	0.74 (0.14)	–
Mobility	–	1 (14.3)	1 (50.0)	6 (60.0)	6 (75.0)	14 (51.9)	–
Self-care	–	1 (14.3)	1 (50.0)	5 (50.0)	3 (37.5)	10 (37.0)	–
Usual activity	–	0 (0.0)	1 (50.0)	4 (40.0)	3 (37.5)	8 (29.6)	–
Pain/discomfort	–	3 (42.9)	2 (100.0)	7 (70.0)	6 (75.0)	18 (66.7)	–
Anxiety/depression	–	2 (28.6)	2 (100.0)	3 (30.0)	4 (50.0)	11 (40.7)	–
*Primipara*	n = 38	n = 101	n = 128	n = 174	n = 280	n = 721	
EQ-VAS^a^	89.29 (10.54)	87.98 (8.68)	88.05 (8.43)	87.7 (8.60)	87.03 (9.26)	87.62 (8.95)	0.555
EQ index value^b^	0.79 (0.30)	0.89 (0.12)	0.86 (0.13)	0.83 (0.20)	0.81 (0.15)	0.83 (0.17)	< 0.001*
Mobility	9 (23.7)	8 (7.9)	12 (9.4)	39 (22.4)	69 (24.6)	137 (19.0)	< 0.001*
Self-care	5 (13.2)	2 (2.0)	8 (6.3)	21 (12.1)	39 (13.9)	75 (10.4)	0.005*
Usual activity	8 (21.1)	5 (5.0)	21 (16.4)	34 (19.5)	58 (20.7)	126 (17.5)	0.007*
Pain/discomfort	17 (44.7)	39 (38.6)	55 (43.0)	77 (44.3)	158 (56.4)	346 (48.0)	0.007*
Anxiety/depression	10 (26.3)	27 (26.7)	42 (32.8)	48 (27.6)	96 (34.3)	223 (30.9)	0.432
*Multipara*	n = 11	n = 13	n = 47	n = 72	n = 44	n = 187	
EQ-VAS^a^	90.91 (8.89)	82.69 (13.17)	89.04 (10.30)	89.33 (9.94)	88.89 (8.18)	88.79 (9.89)	0.223
EQ index value^b^	0.81 (0.34)	0.9 (0.13)	0.87 (0.14)	0.85 (0.17)	0.84 (0.15)	0.86 (0.17)	0.544
Mobility	3 (27.3)	1 (7.7)	5 (10.6)	17 (23.6)	15 (34.1)	42 (22.5)	0.057
Self-care	2 (18.2)	0 (0.0)	2 (4.3)	10 (13.9)	6 (13.6)	20 (10.7)	0.247
Usual activity	1 (9.0)	0 (0.0)	3 (6.4)	14 (19.4)	12 (27.3)	30 (16.0)	0.028*
Pain/discomfort	4 (36.4)	5 (38.5)	18 (38.3)	27 (37.5)	18 (41.0)	72 (38.5)	0.997
Anxiety/depression	4 (36.4)	3 (2.3)	12 (25.5)	15 (20.8)	12 (27.3)	56 (29.9)	0.812
*Non-smoking partner*	n = 44	n = 97	n = 146	n = 208	n = 278	n = 773	
EQ-VAS^a^	89.27 (10.60)	87.4 (9.63)	87.89 (9.18)	87.88 (9.21)	87.45 (9.18)	87.75 (9.32)	0.797
EQ index value^b^	0.78 (0.32)	0.89 (0.12)	0.86 (0.14)	0.83 (0.19)	0.82 (0.15)	0.84 (0.17)	< 0.001*
Mobility	12 (27.3)	6 (6.2)	13 (8.9)	48 (23.1)	71 (25.5)	150 (19.4)	< 0.001*
Self-care	7 (15.9)	2 (2.1)	7 (4.8)	27 (13.0)	37 (13.3)	80 (10.3)	0.001*
Usual activity	9 (20.5)	4 (4.1)	21 (14.4)	42 (20.2)	58 (20.9)	134 (17.3)	0.002*
Pain/discomfort	19 (43.2)	38 (39.2)	62 (42.5)	89 (42.8)	152 (54.7)	360 (46.6)	0.018*
Anxiety/depression	13 (29.5)	26 (26.8)	43 (29.5)	51 (24.5)	93 (33.5)	226 (29.2)	0.297
*Smoking partner*	n = 5	n = 17	n = 29	n = 38	n = 46	n = 135	
EQ-VAS^a^	93 (2.74)	87.24 (8.06)	90.48 (7.50)	89.76 (7.89)	86.24 (8.81)	88.52 (8.19)	0.088
EQ index value^b^	0.91 (0.12)	0.88 (0.12)	0.86 (0.13)	0.83 (0.19)	0.81 (0.13)	0.84 (0.15)	0.394
Mobility	0 (0.0)	3 (17.6)	4 (3.4)	8 (21.1)	13 (28.3)	28 (20.7)	0.428
Self-care	0 (0.0)	0 (0.0)	3 (10.3)	4 (10.5)	9 (19.6)	16 (11.9)	0.222
Usual activity	0 (0.0)	1 (5.9)	3 (10.3)	6 (15.8)	12 (26.1)	22 (16.3)	0.177
Pain/discomfort	2 (40.0)	6 (35.3)	11 (37.9)	15 (39.5)	24 (52.2)	58 (43.0)	0.643
Anxiety/depression	1 (20.0)	4 (23.5)	11 (37.9)	12 (31.6)	15 (32.6)	43 (31.9)	0.849
*Sober partner*	n = 48	n = 110	n = 161	n = 223	n = 291	n = 833	
EQ-VAS^a^	89.65 (10.23)	87.35 (9.52)	88.4 (9.18)	88.35 (8.69)	87.27 (9.26)	87.93 (9.19)	0.343
EQ index value^b^	0.79 (0.31)	0.89 (0.12)	0.86 (0.14)	0.83 (0.20)	0.82 (0.15)	0.84 (0.17)	< 0.001*
Mobility	12 (25.0)	8 (7.3)	15 (9.3)	51 (22.9)	74 (25.4)	160 (19.2)	< 0.001*
Self-care	7 (14.6)	2 (1.8)	9 (5.6)	28 (12.6)	41 (14.1)	87 (10.4)	< 0.001*
Usual activity	9 (18.8)	5 (4.5)	23 (14.3)	43 (19.3)	60 (20.6)	140 (16.8)	0.002*
Pain/discomfort	20 (41.7)	42 (38.2)	67 (41.6)	93 (41.7)	157 (54.0)	379 (45.5)	0.010*
Anxiety/depression	13 (27.1)	29 (26.4)	49 (30.4)	56 (25.1)	94 (32.3)	241 (28.9)	0.434
*Drunk partner*	n = 1	n = 4	n = 14	n = 23	n = 33	n = 75	
EQ-VAS^a^	90 (–)	88.25 (4.72)	87.36 (5.83)	86.43 (11.91)	87.33 (7.98)	87.15 (8.77)	0.988
EQ index value^b^	0.74 (–)	0.87 (0.15)	0.84 (0.11)	0.84 (0.14)	0.79 (0.11)	0.82 (0.13)	0.431
Mobility	0 (0.0)	1 (25.0)	2 (14.3)	5 (21.7)	10 (30.3)	18 (24.0)	0.768
Self-care	0 (0.0)	0 (0.0)	1 (7.1)	3 (13.0)	4 (12.1)	8 (10.7)	0.911
Usual activity	0 (0.0)	0 (0.0)	1 (7.1)	5 (21.7)	10 (30.3)	16 (21.3)	0.329
Pain/discomfort	1 (100.0)	2 (50.0)	6 (42.9)	11 (47.8)	19 (57.6)	39 (52.0)	0.741
Anxiety/depression	1 (100.0)	1 (25.0)	5 (35.7)	7 (30.4)	14 (42.4)	28 (37.3)	0.594
*Cardiovascular* diseases	n = 0	n = 2	n = 1	n = 2	n = 2	n = 7	
EQ-VAS^a^	–	87.5 (10.61)	90 (–)	85 (14.14)	60 (28.28)	79.29 (19.02)	–
EQ index value^b^	–	0.86 (0.19)	0.69 (–)	0.82 (0.26)	0.66 (0.22)	0.77 (0.19)	–
Mobility	–	1 (50.0)	1 (100.0)	1 (50.0)	1 (50.0)	4 (57.1)	–
Self-care	–	0 (0.0)	0 (0.0)	0 (0.0)	1 (50.0)	1 (14.3)	–
Usual activity	–	0 (0.0)	1 (100.0)	1 (50.0)	1 (50.0)	3 (42.9)	–
Pain/discomfort	–	1 (50.0)	1 (100.0)	1 (50.0)	2 (100.0)	5 (71.4)	–
Anxiety/depression	–	0 (0.0)	0 (0.0)	1 (50.0)	1 (50.0)	2 (28.6)	–
*Hepatitis B*	n = 1	n = 3	n = 2	n = 3	n = 10	n = 19	
EQ-VAS^a^	100 (–)	95 (5.00)	92.5 (10.61)	91.67 (2.89)	84.8 (10.51)	89.11 (9.50)	0.302
EQ index value^b^	0.81 (–)	0.82 (0.01)	1 (0.00)	0.86 (0.13)	0.85 (0.14)	0.86 (0.12)	0.554
Mobility	0 (0.0)	0 (0.0)	0 (0.0)	0 (0.0)	1 (10.0)	1 (5.3)	0.917
Self-care	0 (0.0)	0 (0.0)	0 (0.0)	1 (33.3)	0 (0.0)	1 (5.3)	0.229
Usual activity	0 (0.0)	0 (0.0)	0 (0.0)	1 (33.3)	2 (20.0)	3 (15.8)	0.744
Pain/discomfort	1 (100.0)	2 (66.7)	2 (100.0)	1 (33.3)	4 (40.0)	10 (52.6)	0.403
Anxiety/depression	0 (0.0)	1 (33.3)	0 (0.0)	0 (0.0)	5 (50.0)	6 (31.6)	0.361
*GDM*^*c*^	n = 3	n = 12	n = 23	n = 33	n = 49	n = 120	
EQ-VAS^a^	88.33 (16.07)	85 (13.48)	87.83 (11.36)	85.97 (10.42)	88.84 (9.71)	87.46 (10.69)	0.717
EQ index value^b^	0.75 (0.07)	0.91 (0.14)	0.86 (0.15)	0.82 (0.14)	0.85 (0.14)	0.85 (0.14)	0.254
Mobility	2 (66.7)	3 (25.0)	4 (17.4)	12 (36.4)	10 (20.4)	31 (25.8)	0.189
Self-care	0 (0.0)	0 (0.0)	1 (4.3)	2 (6.1)	3 (6.1)	6 (5.0)	0.907
Usual activity	1 (33.3)	0 (0.0)	5 (21.7)	8 (24.2)	8 (16.3)	22 (18.3)	0.377
Pain/discomfort	2 (66.7)	4 (33.3)	9 (39.1)	16 (48.5)	24 (49.0)	55 (45.8)	0.739
Anxiety/depression	1 (33.3)	2 (16.7)	8 (34.8)	13 (39.4)	14 (28.6)	38 (31.7)	0.648
*Scarred uterus*	n = 13	n = 23	n = 36	n = 63	n = 84	n = 219	
EQ-VAS^a^	88.85 (11.39)	87.61 (8.24)	90.36 (8.42)	89.38 (7.64)	89.21 (8.04)	89.26 (8.19)	0.806
EQ index value^b^	0.8 (0.22)	0.87 (0.13)	0.89 (0.14)	0.86 (0.13)	0.84 (0.16)	0.86 (0.15)	0.385
Mobility	6 (46.2)	5 (21.7)	4 (11.1)	16 (25.4)	19 (22.6)	50 (22.8)	0.132
Self-care	1 (7.7)	1 (4.4)	0 (0.0)	4 (6.3)	7 (8.3)	14 (6.4)	0.504
Usual activity	1 (7.7)	3 (13.0)	4 (11.1)	14 (22.2)	18 (21.4)	40 (18.3)	0.427
Pain/discomfort	6 (46.2)	9 (39.1)	9 (25.0)	22 (34.9)	38 (45.2)	84 (38.4)	0.282
Anxiety/depression	5 (38.5)	6 (26.1)	8 (22.2)	13 (20.6)	22 (26.2)	54 (24.7)	0.712
*Non-complications *^*d*^	n = 25	n = 78	n = 99	n = 85	n = 103	n = 390	
EQ-VAS^a^	87.4 (12.68)	86.74 (10.01)	89.55 (8.71)	88.32 (8.48)	88.37 (9.13)	88.27 (9.34)	0.386
EQ index value^b^	0.7 (0.39)	0.89 (0.12)	0.87 (0.14)	0.8 (0.27)	0.83 (0.13)	0.83 (0.20)	< 0.001*
Mobility	8 (32.0)	6 (7.7)	9 (9.0)	17 (20.0)	27 (26.2)	67 (17.2)	< 0.001*
Self-care	7 (28.0)	2 (2.6)	5 (5.5)	13 (15.3)	14 (13.6)	41 (10.5)	< 0.001*
Usual activity	6 (24.0)	5 (6.4)	10 (10.1)	17 (20.0)	19 (18.4)	57 (14.6)	0.029*
Pain/discomfort	12 (48.0)	31 (39.7)	37 (37.4)	42 (49.4)	52 (50.5)	174 (44.6)	0.268
Anxiety/depression	10 (40.0)	22 (28.2)	31 (31.1)	22 (25.9)	30 (29.1)	115 (29.5)	0.720

Reported problem of each dimension: EQ-5D level 2–5; One-Way Analysis of Variance (ANOVA) test was used to calculated the EQ-VAS and EQ index value. Chi square test was used to calculated the reported problems (percentage) in EQ-5D dimensions

*P value < 0.05 indicates the statistical difference

^a^EuroQol-visual analogue scale

^b^EuroQol index value

^c^Gestational diabetes mellitus

^d^Including thyroid diseases, thalassemia, obesity, etc.

**Table 3:** Frequency (percentage) of reporting levels 1 to 5 in EQ-5D dimensions across five gestational stages

EQ-5D DIMENSION	Trimester-specific					Overall	*P* value
	1st	Early 2nd	Late 2nd	Early 3rd	Late 3rd	n = 908	
	n = 49	n = 114	n = 175	n = 246	n = 324		
*Mobility*							< 0.001*
Level 1	37 (75.5)	105 (92.1)	158 (90.3)	190 (77.2)	240 (74.1)	730 (80.4)	
Level 2	7 (14.3)	9 (7.9)	15 (8.6)	45 (18.0)	74 (22.7)	150 (16.6)	
Level 3	0 (0.0)	0 (0.0)	0 (0.0)	4 (1.6)	7 (2.2)	11 (1.2)	
Level 4	1 (2.0)	0 (0.0)	0 (0.0)	1 (0.4)	1 (0.0)	3 (0.3)	
Level 5	4 (8.2)	0 (0.0)	2 (1.1)	6 (2.4)	2 (1.0)	14 (1.5)	
Reported problem	12 (24.5)	9 (7.9)	17 (9.7)	56 (22.8)	84 (25.9)	178 (19.6)	
*Self-care*							< 0.001*
Level 1	42 (85.7)	112 (98.2)	165 (94.3)	215 (87.4)	279 (86.1)	813 (89.5)	
Level 2	2 (4.1)	2 (1.8)	10 (5.7)	24 (9.8)	39 (11.9)	77 (8.5)	
Level 3	0 (0.0)	0 (0.0)	0 (0.0)	1 (0.4)	2 (1.0)	3 (0.3)	
Level 4	0 (0.0)	0 (0.0)	0 (0.0)	0 (0.0)	1 (0.0)	1 (0.2)	
Level 5	5 (10.2)	0 (0.0)	0 (0.0)	6 (2.4)	3 (1.0)	14 (1.5)	
Reported problem	7 (14.3)	2 (1.8)	10 (5.7)	31 (12.6)	45 (13.9)	95 (10.5)	
*Usual activity*							< 0.001*
Level 1	40 (81.6)	109 (95.6)	151 (86.3)	198 (80.5)	254 (78.4)	752 (82.8)	
Level 2	4 (8.2)	5 (4.4)	24 (13.7)	38 (15.4)	61 (19.1)	132 (14.5)	
Level 3	0 (0.0)	0 (0.0)	0 (0.0)	3 (1.2)	5 (1.5)	8 (0.9)	
Level 4	1 (2.0)	0 (0.0)	0 (0.0)	3 (1.2)	3 (1.0)	7 (0.8)	
Level 5	4 (8.2)	0 (0.0)	0 (0.0)	4 (1.6)	1 (0.0)	9 (1.0)	
Reported problem	9 (18.4)	5 (4.4)	24 (13.7)	48 (19.5)	70 (21.6)	156 (17.2)	
*Pain/discomfort*							< 0.001*
Level 1	28 (57.1)	70 (61.4)	102 (58.3)	142 (57.7)	148 (45.7)	490 (54.0)	
Level 2	15 (30.6)	44 (38.6)	71 (40.6)	90 (36.6)	166 (51.2)	386 (42.5)	
Level 3	1 (2.0)	0 (0.0)	2 (1.1)	7 (2.8)	8 (3.1)	18 (2.0)	
Level 4	0 (0.0)	0 (0.0)	0 (0.0)	3 (1.2)	2 (0.0)	5 (0.6)	
Level 5	5 (10.2)	0 (0.0)	0 (0.0)	4 (1.6)	0 (0.0)	9 (1.0)	
Reported problem	21 (42.9)	44 (38.6)	73 (41.7)	104 (42.3)	176 (54.3)	418 (46.0)	
*Anxiety/depression*							< 0.001*
Level 1	35 (71.4)	84 (73.7)	121 (69.1)	183 (74.4)	216 (66.7)	639 (70.4)	
Level 2	9 (18.4)	29 (25.3)	48 (27.4)	53 (21.5)	100 (30.8)	239 (26.3)	
Level 3	0 (0)	1 (1.0)	5 (2.9)	1 (0.4)	5 (1.5)	12 (1.3)	
Level 4	1 (2.0)	0 (0.0)	0 (0.0)	4 (1.6)	3 (1.0)	8 (0.9)	
Level 5	4 (8.2)	0 (0)	1 (1.0)	5 (2.0)	0 (0.0)	10 (1.1)	
Reported problem	14 (28.6)	30 (26.3)	54 (30.9)	63 (25.6)	108 (33.3)	269 (29.6)	

Reported problem: EQ-5D level 2–5; Chi square test was used to calculated the data

*P value < 0.05 indicates the statistical difference

## Results

A total of 908 pregnant women completed the EQ-5D-5L questionnaire at least once, and 908 questionnaires were compiled and included for further analysis. Table 1 listed the baseline characteristics and the mean and standard deviation of EQ-VAS score of 908 pregnant women. Across the gestational stages, the mean EQ-VAS was highest in the 1st trimester (89.65 ± 10.13) and lowest in the late 3rd trimester (87.28 ± 9.13). The mean EQ index value for each consecutive pregnancy trimester was 0.79 ± 0.31, 0.89 ± 0.12, 0.86 ± 0.13, 0.83 ± 0.19, and 0.82 ± 0.15 (*P* value < 0.05), respectively.

Table 2 shows the EQ-VAS and EQ index value of pregnant women in different conditions, with or without complications. There are 3,125 (equals 5 to the power of 5) types of possible response patterns. Among 908 questionnaires in this study, 72 types of patterns occurred at least once. 39.1% of our sample (n = 355) reported the optimal response pattern ‘11,111’, which meaned these participants had no problems on all these five dimensions. Regardless of the conditions, the dimension with the most problems for pregnant women was pain/discomfort. Moreover, compared with other gestational stages, pregnant women in late 3rd trimester reported more problems in each dimension.

The response frequencies for each of the five dimensions, classified by gestational stages (i.e., 1st, early 2nd, late 2nd, early 3rd, and late 3rd trimesters) were listed in Table 3. Roughly one fifth (19.6%) of responders had health-related problems (levels 2–5) related to mobility, 10.5% had problems related to self-care, 17.2% had problems with usual activity, 46.0% had problems related to pain/discomfort, and 29.6% had problems related to anxiety/depression.

Moreover, the profile of women who reported having “problems” is presented in Table 3. As noted in Table 3, problems related to pain/discomfort were the most common (46.0%); problems related to self-care were the least common (10.5%). Furthermore, more than 20% of women had problems in one of the five dimensions, except self-care, in the 1st trimester. Pregnant women who reported having problems related to mobility, usual activity and pain/discomfort were the most common during the late 3rd trimester. In contrast, problems related to self-care and anxiety/depression occurred the most frequently during the 1st trimester.

**Fig. 1:**
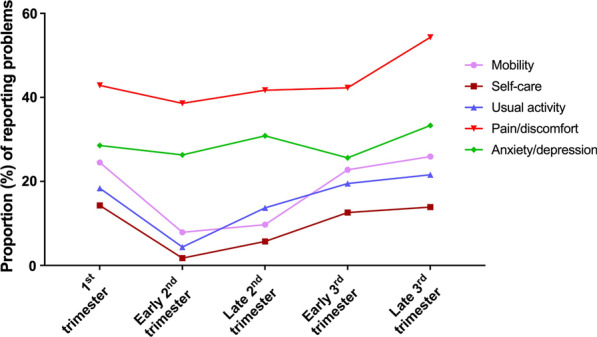
Profile of the proportion (%) with problems by dimension and gestational period

As shown in Fig. 1, the proportion of women reporting problems in mobility, self-care and usual activity fell significantly from the 1st trimester to the early 2nd trimester and then increased again with gestational age. Meanwhile, there was a slight decrease from the 1st trimester to the early 2nd trimester in the proportion of reporting problems with pain/discomfort, and then increased significantly in later stages. Nevertheless, there was no evident change in proportion of women who reported problems with anxiety/depression.

**Fig. 2:**
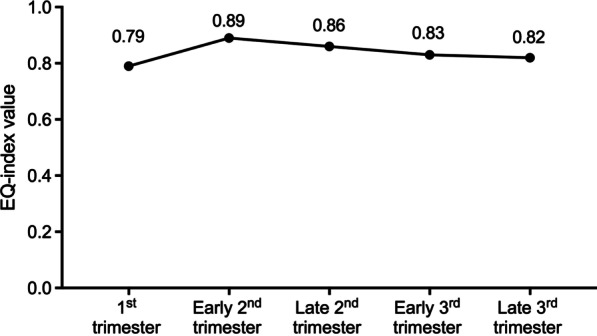
Fluctuation of the EQ index value with gestational age

Fluctuations in EQ index value with gestational age were shown in Fig. 2. We observed an increasing in EQ index value from the 1st to early 2nd trimester and gradual decreases thereafter.

## Discussion

Our study showed that HRQoL of pregnant women was the highest in the early 2nd trimester and reduced gradually at later times during pregnancy. The results were consistent with those of other studies related to the HRQoL of pregnant women. Haas et al. (2005) suggested that health status declined substantially during pregnancy, and then improved after delivery [29]. Sut et al. [20] found that found that sleep quality and HRQoL during pregnancy had close relationship, and EQ index scores significantly decreased in the 2nd and 3rd trimesters compared with the 1st trimester. However, to the best of our knowledge, no study has yet investigated how HRQoL changes with the increasing of gestational age in China. Our study also analysed the EQ index and found there was a relationship between five dimensions (mobility, self-care, usual activities, pain/discomfort, anxiety/depression) in pregnant women and different gestational ages.

Early in the pregnancy, pregnant women may experience a lower HRQoL due to severe morning sickness, severe vomiting or fear of fetal loss among others. In the early 2nd trimester, HRQoL was at its highest in our study. However, with increasing gestational age, women begin to experience more physical and psychological changes, including enlarged uterine, gain of weight, poor sleep quality, etc. [30, 31]. At later stages of pregnancy, problems arise with mobility, self-care, and daily activity due to the progressive distension of the belly and the associated inconvenience. Furthermore, some pregnant women experience additional physical discomfort, such as pelvic pain and chest distress [32], as a direct result of the enlargement of the uterus. Moreover, complications, fear of childbirth, and the impact of pregnancy on sexual life may elevate anxiety and depression. Therefore, during the late 3rd trimester, pregnant women would face majority of the problems covered by the five dimensions of the EQ-5D-5L questionnaire and this may explain the low HRQoL we observed in the 3rd trimester compared with the early 2nd trimester.

On the other hand, pregnant women who reported having problems with anxiety/depression remained relatively constant throughout the duration of pregnancy and always exceeded 25%, and problems with anxiety/depression of pregnant women was minimally influenced by gestational age. A possible reason for this trend is that the anxiety/depression of pregnant women is primarily caused by objective factors such as first time pregnancy [33], and socioeconomic status [34, 35]. Moreover, physiological fluctuation of estrogen during pregnancy can also affect emotional status, leading to anxiety and depression in pregnant women [36], all of these are weakly related to gestational age associated changes.

More than 15% of pregnant women reported problems in the 1st trimester (i.e., EQ-5D-5L levels 2–5), which seemed contradictory to our result that the HRQoL of pregnant women during the 1st trimester was the second highest, surpassed only by that early in the 2nd trimester. A possible explanation for this finding was that majority of pregnant women who reported problems on their EQ-5D questionnaires had only slight problems, which resulted in a relatively high average EQ index value. Overall, the HRQoL of pregnant women during the 1st trimester was relatively better. Interestingly, the EQ index of women with Gestational Diabetes Mellitus (GDM) was relatively higher than normal pregnant women. According to the guidelines of diabetes in pregnancy from American Diabetes Association and National Institute for Health and Care Excellence in England, more clinical attention should be paid for pregnant women with GDM during regular antenatal care such as diabetic education, blood glucose monitoring and pharmacologic therapy [37, 38]. This might be a possible reason why these pregnant women had relatively higher EQ index.

Evaluating HRQoL is becoming increasingly important in healthcare due to the cost-effectiveness of medical decisions. Due to the complexity of pregnancy, medical decisions can be challenging. HRQoL measured by EQ-5D can assist clinicians in better understanding the changes of pregnant women in different trimesters and inform clinical decision-making and resource allocation. Thus, nursing practitioners can provide relevant nursing and education for pregnant women more purposefully, helping them have a higher quality of pregnancy and better pregnancy outcomes, such as guiding them to deal with physiological changes and releasing anxiety and depression. Therefore, evaluating HRQoL can provide a new focus for future antenatal care for women to obtain better care during their whole pregnancy. In addition, the changes in HRQoL of pregnant women in different pregnancy periods can be monitored to have more precise management for pregnant women in line with the needs of women’s nursing and caring strategies. Besides, HRQoL can be used as an indicator to assist in medical decision-making during pregnancy. HRQoL has been commonly used in health policy research, as it effectively assesses HRQoL among different populations [39–41], and our study can provide a reference HRQoL value for the pregnancy populations. Our study found that the average value of the EQ index in our study population is 0.84; one of the possible uses of this reference value is that if a Chinese woman scores one SD (− 0.17) lower than the average, that could be an indicator to provide more attention regarding their antenatal care. In addition, our data could provide a reference for another similar setting in Asia and other parts of the world. However, it is strongly recommended to use EQ-5D to evaluate the local HRQoL of pregnancy populations in a different country.

## Strengths and limitations

The main strength of this study is to focus on HRQoL in pregnant women with different gestational age, especially focus on the comparison between different trimester. In addition, different variables such as smoking or drinking status of partners contributes to the comprehensive understanding and comparisons of HRQoL of pregnant women in different gestational age. Moreover, it can provide utility-based case values in pregnant women with different gestational ages in clinic and they may contribute to health economic studies. Nevertheless, our study has some limitations. Firstly, this study is a cross-sectional study, which cannot provide the longitudinal changes in the HRQoL of pregnant women in different trimesters. Moreover, when we analysed how HRQoL of pregnant women changed with the increasing of gestational age, it was unable to exclude the effect of complications prior to and during pregnancy on HRQoL. However, they may have major impact on the HRQoL. In further studies, subgroup analysis and statistical stratifications will be necessary to clarify the contribution of complications or medical conditions in pregnancy. Secondly, our results may not be comprehensive due to some missing data regarding fetal loss, pregnant women who refused to participate, and other reasons that led to follow-up loss or sample gaps. Further studies should bridge these gaps by including data on miscarriage, the number of previous successful (and unsuccessful) pregnancies, maternal education and financial situations. Thirdly, the applicability of our results may be limited, because pregnant women from one regional university hospital may not be reflective of all pregnant women in China. Thus, data from multi-central trials would be more representative.

## Conclusions

In our study, it was found that HRQoL of pregnant women was the highest in the early 2nd trimester and then decreased to the lowest in the late 3rd trimester due to a series of physical and psychological changes. Our study provides some utility-based case values in pregnant women with different gestational ages in different conditions such as obstetrics complications. These can provide basis of HRQoL data and guide for cost-utility analyses and health economic studies in the future. Moreover, obstetric doctors and medical institutions should provide more antenatal care to pregnant women and help them to better face the series of changes during the whole pregnant period.

## Data Availability

The datasets used and/or analyzed during the current study are available from the corresponding author on reasonable request.

## Abbreviations

HRQoL: Health-related quality of life
EQ-5D-5L: The EuroQoL Group’s five-dimension five-level questionnaire
EQ-VAS: EuroQoL Group’s visual analog scale questionnaire
BMI: Body mass index
SD: Standard deviation
IQR: Interquartile range
GDM: Gestational Diabetes Mellitus
